# The effect of a Clinical Decision Support System on the frequency of dose adjustments of anticancer drugs in case of renal or hepatic dysfunction

**DOI:** 10.1177/10781552211019453

**Published:** 2021-06-10

**Authors:** José Rolvink, Anne-Loes E Gerards, Arnon P Kater, Eleonora L Swart, Matthijs L Becker

**Affiliations:** 1 University of Amsterdam, Amsterdam, the Netherlands; 2 Pharmacy Foundation of Haarlem Hospitals, Haarlem, the Netherlands; 3 Spaarne Gasthuis, Haarlem/Hoofddorp, the Netherlands; 4 Northwest Clinics, Alkmaar, The Netherlands

**Keywords:** Clinical decision support system, CDSS, Antineoplastic agents, renal dysfunction, hepatic dysfunction, oncology, dose adjustment

## Abstract

**Introduction:**

Dose adjustments in patients with renal or hepatic dysfunction using anticancer drugs are indicated according to guidelines. However, implementation depends on awareness of prescribing physicians. We implemented a Clinical Decision Support System (CDSS), recommending dose adjustments upon electronic prescriptions based on renal and hepatic function. The alert provides a dose adjustment proposal and recent laboratory results. Our objective was to determine the frequency of dose adjustments before and after implementation of this CDSS.

**Methods:**

We included all first orders for patients ≥18 years treated with parenteral antineoplastic agents, for whom dosage adjustment is necessary based on renal or hepatic function between February 2018 and January 2019. This study was performed at the department of Clinical Oncology and Hematology of the Amsterdam University Medical Center. We implemented the CDSS August first. All prescriptions were prescribed according to common practice. We analyzed the orders where a dose reduction based on renal or hepatic function was indicated.

**Results:**

We included 73 orders before implementation and 99 orders after implementation. Before implementation 21% of doses were reduced in line with the guidelines versus 34% after implementation (p = 0.048). For hepatic dysfunction the proportion changed from 11% to 46% p = 0.011, while there was no effect for renal dysfunction (24% vs. 26% p = 0.75).

**Conclusion:**

Dosages are more frequently adjusted in concordance with guidelines in patients with hepatic dysfunction who are treated with parenteral antineoplastic agents after implementation of a CDSS. The change was not seen in patients with renal dysfunction.

## Introduction

Dose adjustment of antineoplastic agents in patients with renal or hepatic dysfunction are recommended to prevent toxicity.^1–3^ Guidelines for these dose adjustments are given by the manufacturer and can be found in literature and protocols.^4–8^ Over the last decades, the average age of cancer patients has increased due to the aging population and expansion of available therapies. Therefore renal or hepatic dysfunction is a more frequent comorbidity in cancer patients.^1,2,8^ The Renal Insufficiency and Cancer Medication (IRMA) studies showed a prevalence of around 50% for renal dysfunction prior to treatment.^1,2^ This is associated with a increased mortality after 2 years, with a hazard ratio of 1.27.^9^ The reduced survival might be the consequence of cardiovascular complications associated with renal dysfunction or related to the anticancer therapy.^1,2,9,10^

Prevalence data of hepatic dysfunction in cancer patients is, to the best of our knowledge, not available. Dose adjustment in hepatic dysfunction may be necessary for two reasons. Firstly, hepatic dysfunction can lead to a reduced metabolism of the anticancer drugs and thus a higher exposure. Secondly, antineoplastic agents themselves can induce liver injury.^8,11,12^

**Figure 1.**
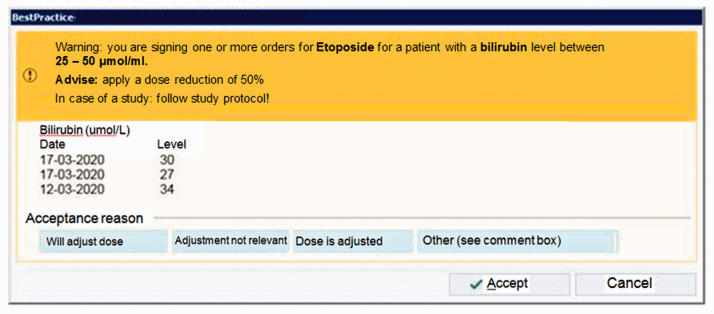
Example of the pop-up as shown at the prescriber. With selecting ‘Accept’ the order is signed as originally prescribed. With selecting ‘Cancel’ the prescriber returned to the order and the dose could be altered.

In our hospital, we implemented a Clinical Decision Support System (CDSS) for parenteral antineoplastic agents in patients with renal or hepatic dysfunction. It generates a pop-up alert shown to the prescriber with a patient specific recommendation for dose adjustment and the three most recent laboratory results for renal or hepatic function (Figure 1). We studied whether the implementation resulted in more frequent dose adjustments in patients with renal or hepatic dysfunction using antineoplastic agents.

## Material and methods

### Organizational setting

This study was initiated by the Amsterdam UMC, University of Amsterdam (Amsterdam, the Netherlands), a university hospital with two locations; Amsterdam Medical Center (AMC) and VU Medical Center (VUMC). The study was performed on both locations.

### Study design

We performed a prospective intervention study, in which we compared the frequency of dose adjustments before and after the implementation of the alert. The recommendations in the alerts were compiled from the summary of product characteristics, BC cancer and Up-to-Date.^4–8^ The antineoplastic agents listed in Table 1 with accompanying dose adjustments were analyzed. For renal and hepatic dysfunction standard protocols of the Amsterdam UMC referred to BC cancer for dose adjustments.^4^

**Table 1.** Selected antineoplastic agents and advised dose adjustment for renal or hepatic dysfunction.

Antineoplastic agent	eGFR (ml/min)	Advised reduction (%)	Bilirubin (µmol/L)	Advised reduction (%)	References
Bendamustine	–	–	20–50 >50	30 Avoid use	5,7,8
Bleomycin	10–50 <10	25 50	–	–	4,8
Bortezomib	–	–	>50	50	4,5
Cisplatin	45–60 <45	25 Avoid use	–	–	4
Cyclophosphamide	<10	25	50–85 >85	25 Avoid use	4,5,7
Cytarabine	45–60 30–45	40 50	>34	50	4,6,7
Daunorubicin	–	–	25–50 50–85 >85	25 50 Avoid use	4,7
Doxorubicin^a^	–	–	25–50 50–85 >85	50 75 Avoid use	4,5,8
Epirubicin	–	–	20–50 >50	50 75	4,5,7,8
Etoposide	10–50 <10	25 50	25–50 50–85 >85	50 75 Avoid use	4,5,7
Fludarabine	30–50 <30	50 Avoid use	–	–	4,5
Idarubicin	10–50 <10	25 50	25–50 50–85 >85	25 50 Avoid use	4
Ifosfamide	<10	25	–	–	4
Irinotecan	–	–	30–50	25	5,7
Melphalan	10–50 <10	25 50	–	–	4,5
Methotrexate	10–50 <10	50^b^ Avoid use	50–85 >85	25 Avoid use	4,7
Oxaliplatin	<30	Avoid use	–	–	4,5
Pemetrexed	<45	Avoid use	–	–	4,5
Topotecan	20–40 <20	50 Avoid use	–	–	4,5
Vinblastine	–	–	25–50 >50	50 Avoid use	4,5
Vincristine	–	–	25–50 >50	50 Avoid use	4,5

The advised reduction is the percentage with which de dosage should be reduced in relation to the standard dose of the treatment protocol.

^a^Does not apply to doxorubicin liposomal.

^b^High dose methotrexate in combination with therapeutic drug monitoring (TDM).

Because the implementation of the alert was part of improving regular care, no approval of an ethical committee was needed.

### Population

We included all first orders for antineoplastic agents listed in Table 1 from the moment a dose adjustment was indicated. Only the first order from the moment a dose adjustment was indicated was included in the analysis, and the second and subsequent orders for the same agent within one treatment plan for which dose adjustment was applicable were excluded. Drugs with dosing recommendations for both renal and hepatic dysfunction were analyzed in both analyses. For instance; during treatment with R-CHOP, cyclophosphamide was analyzed both for renal and hepatic dysfunction. Doxorubicin and vincristine were analyzed in case of liver dysfunction only. A total of four orders from one patient receiving R-CHOP could be included during the study if a patient had both renal and hepatic dysfunction. Orders from both inpatients and outpatients were included. Patients included in the pre-intervention phase were excluded from the post-intervention phase.

Orders for patients aged < 18 years, doxorubicin liposomal, fixed dose doxorubicin or cyclophosphamide, individually dosed methotrexate were excluded. Combined infusion of etoposide, vincristine and doxorubicin in one infusion bag are adjusted conform specific dose levels in the protocol and therefore not included.

### Study flow

We implemented the alert on 1 August 2018 in the hospital information system Epic (Epic, Verona, WI). We analyzed the 6 months before implementation as a historical control group (1 February 2018 until 31 July 2018) and the 6 months after implementation as intervention group (1 August 2018 until 31 January 2019). Before implementation a generic alert was shown to the physician in case of renal dysfunction. A generic advice was given to reconsider dosage, dosing frequency or use of the agent, without mentioning the recommended percentage of dose reduction. No alert was shown in case of hepatic dysfunction. During the intervention period, a patient specific advice was shown to the physician, with the recommended percentage of dose reduction for this patient including the three most recent laboratory results for renal or hepatic function. In both groups the orders were checked by a pharmacist before preparation of the anticancer drug. When needed a dose adjustment was suggested to the physician. Guidelines for dosage reductions in renal dysfunction are based on the estimated glomerular filtration rate (eGFR) and in our center the CKD-EPI (Chronic Kidney Disease Epidemiology Collaboration) formula is used to calculate the eGFR. Guidelines for dosage reductions in hepatic dysfunction are most frequently based on bilirubin. Therefore we choose the bilirubin as a parameter for hepatic dysfunction.

An example of this alert is shown in Figure 1. The alert was shown to the physician at the moment the order was signed.

### Methods for data acquisition

Data acquisition was performed using SQL queries to extract all relevant information from Epic. For the period after implementation, information on the number of alerts and the handling of the alert was collected.

### Outcome and statistical analysis

The endpoint was the frequency of dose adjustments for orders that needed to be reduced for renal or hepatic dysfunction in concordance with Table 1. We stated an order as adjusted when the dose was adjusted with the recommended percentage or more. The difference in frequency of dose adjustments in the control group and intervention group is tested using the Pearson’s Chi-square test. Sub analyses were performed for renal and hepatic dysfunction, for solid and hematological tumors and for hospital location (AMC and VUMC). In the intervention group, the number and handling of the alerts was analyzed. All analyses were performed using SPSS version 25.0 Software (IBM Corp., Version 25.0. Armonk, NY).

## Results

**Figure 2.**
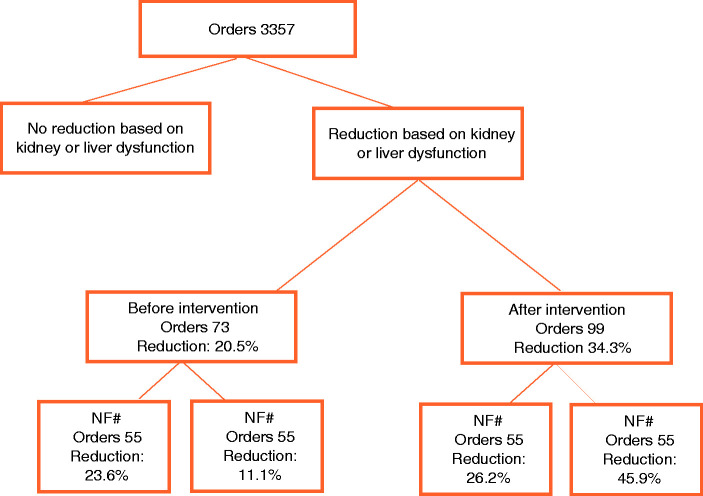
Flow diagram of included orders. The total amount of orders represent all first orders per patient, treatment plan and agent.

A total of 1499 orders for the antineoplastic agents in Table 1 were prescribed before implementation of the alert and 1858 orders after implementation. We included 172 first orders (5.1%) for which dose adjustment was indicated based on kidney or hepatic dysfunction (Figure 2 and Table 2).

**Table 2.** Population characteristics.

	Pre-intervention	Intervention
Orders where dose adjustment is necessary	73	99
Patients	58	77
Gender	♂43 (59%)	♂52 (53%)
Age	61 years (SD ±13)	60 years (SD ±14)
Number of included agents in a treatment plan	1 agent: 47	1 agent: 57
	2 agents: 7	2 agents: 18
	3 agents: 4	3 agents: 2
Orders adjusted for renal dysfunction	55 (75 %)	62 (63%)
Hematologic malignancy	47 (64%)	64 (65%)
Location site	17 AMC (23%)	40 AMC (40%)
Kidney function^a^	– eGFR: 45 ml/min/1.73m^2^ (SD ± 12)	– eGFR: 43 ml/min/1.73m2 (SD ± 13)
	– creatinine: 1.6 mg/dl(SD ± 0.6)	– creatinine: 1.8 mg/dl (SD ± 1.5)
Bilirubin^a^	48 µmol/L (SD ± 43)	46 µmol/L (SD ± 32)

^a^The kidney function was only known for agents selected for the kidney function. The bilirubin was only known for agents selected for the hepatic function.

Before implementation of the alert, the dose of 73 orders should be adjusted according to the recommendations in Table 1 and 15 orders (21%) were actually adjusted. After implementation, the dose of 99 orders should be adjusted according to the recommendations in Table 1 and 34 orders (34%) were adjusted. The difference was statistically significant (p = 0.048). No significant difference was seen for dose adjustment in renal dysfunction (24% vs. 26% p = 0.75), while for the dose adjustments in hepatic dysfunction there was a significant difference (11% vs. 46% p = 0.011).

Dose adjustment was independent of tumor type (solid tumors 15% vs. 31% p = 0.15; hematological malignancies 23% vs. 36% p = 0.16) or location site (AMC 23% vs. 36% p = 0.16; VUMC 35% vs. 45% p = 0.50).

The alert was shown 108 times during the implementation period. Four times treatment with the agent was ceased and five times the alert was shown unjustified. Four times methotrexate was prescribed subcutaneous, for which no dose adjustment is needed. One time bleomycin was prescribed as intralesional treatment, where no dose adjustment is needed.

**Figure 3.**
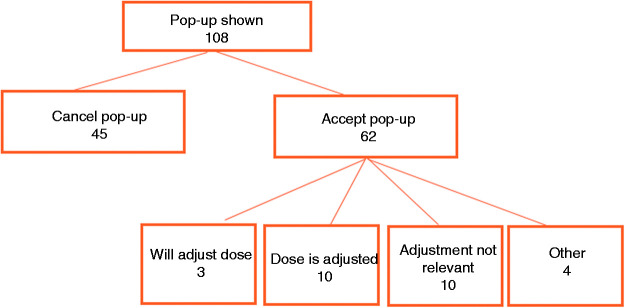
Action taken after the pop-up triggered and subsequent actions.

The alert was 62 times (57%) acknowledged and the order not altered (Figure 3). For 27 of the 62 orders, the physician gave a reason for not changing the order. For 20 of the 27 orders, the physician mentioned that dose adjustment was not relevant or that the dose was already adjusted.

## Discussion

This study shows that before implementation the dose of parenteral antineoplastic agents is adjusted according to guidelines for renal or hepatic dysfunction in only 21% of the cases. After implementation of the alert with a patient specific recommendation, this percentage increases to 34%. For orders in patients with hepatic dysfunction the proportion of dose adjustments increases with 35% from 11 to 46%, while for orders in patients with renal dysfunction no statistically significant difference was seen. The change in percentage of dose reductions was independent of the type of tumor or hospital location.

To the best of our knowledge this is the first study that assessed whether a patient specific alert at the moment of prescribing parenteral antineoplastic agents for patients with renal or hepatic dysfunction increases the adherence to guidelines. Other studies have assessed the effect of the implementation of computerized order entry systems and CDSS’s within the field of oncology, but none of these studies looked specifically at decision support in patients with renal or hepatic dysfunction.^13^

For patients with hepatic dysfunction a significant increase in dose reductions was found after implementation of the alert, while for patients with renal dysfunction no increase was found. Before implementation of the patient specific alert, for patients with renal dysfunction a general alert was already given while for patients with hepatic dysfunction such an alert was not present. It is possible that in patients with hepatic dysfunction, the introduction of the new alert increased awareness for dose adjustments.

Even though we see more dose adjustments, for the majority of patients the dose is not reduced in line with the guidelines. The reasons not to apply a dose adjustment according to guidelines can be versatile; either alert fatigue may play a role or there may be a clinically sound reason not to adjust the dose in line with the recommendations.^14^ The pharmacist proposes a dose adjustment to the prescribing physician if needed, before preparation of the anticancer drug. The alert however is not shown to a pharmacist. For a curative treatment, the physician might accept a certain level of toxicity if this results in a higher chance of treatment success. However for a palliative treatment the enhanced toxicity might not be outweighed by the limited prolonged survival. Another explanation may be that the alert triggers at high levels of bilirubin, while we did not include the cholestatic liver enzymes. If only the bilirubin level is high, but the cholestatic enzymes levels are within normal range, the physician may judge that a dose reduction is not needed because the patient is hepatic sufficient. We choose bilirubin as surrogate parameter for hepatic dysfunction because most guidelines are based on bilirubin and not the cholestatic enzymes.

Furthermore we only analyzed the first order where dose reductions are indicated and it is possible that a dose is not adjusted directly at the first moment of a deviation in eGFR or bilirubin.

Finally we included orders for the treatment of liver tumors. In liver tumors, bilirubin levels may be high but are no reason to reduce the dose of the antineoplastic agent.

Our study has some potential strengths and limitations. A strength of this study is that we were, to the best of our knowledge, the first to assess the effect on patient specific alerts for dose adjustments in patients with renal and hepatic dysfunction. We were able to include a substantial number of orders where dose adjustments should be performed according to the guidelines. A limitation of this study is that we did not have information about the intention of the treatment and could not differentiate between curative and palliative treatments. Also the pop-up triggers at the agent level irrespective of other relevant criteria, such as the dose and indication. For example, cytarabin should only be adjusted when given as high dose, while the toxicity of low dose cytarabin is limited.^7^ At last, we did not assess the effect on clinical outcomes. To assess this association, a much larger study population should be included.

Further improvements could increase the relevancy of the alert. If the pop up would be incorporated in a treatment plan, various limitations could be circumvented. In treatment plans for liver cancer, dose adjustments in case of an increased bilirubin level are not indicated and no alert should fire. Moreover, the unadjusted dose could be used in the treatments plan and the algorithm could be changed that no alert would fire if the dose is already adjusted in line with the guidelines. In general the alert should be made both treatment plan and agent specific to increase de usefulness for the prescriber. Previous handling of the alert could also be incorporated to give insight of earlier reasons to dosage adjustment or to refrain from dosage adjustment.

## Conclusion

Dosages are more frequently adjusted in concordance with guidelines in patients with renal or hepatic dysfunction who are treated with parenteral antineoplastic agents after implementation of a CDSS. The percentage of doses adjusted in line with the guidelines increased from 21 to 34 percent. The increase in dose adjustments is only present for orders prescribed to patients with hepatic dysfunction.

## Supplemental Material

sj-pdf-1-opp-10.1177_10781552211019453 - Supplemental material for The effect of a Clinical Decision Support System on the frequency of dose adjustments of anticancer drugs in case of renal or hepatic dysfunction Click here for additional data file. Supplemental material, sj-pdf-1-opp-10.1177_10781552211019453 for The effect of a Clinical Decision Support System on the frequency of dose adjustments of anticancer drugs in case of renal or hepatic dysfunction by José Rolvink, Anne-Loes E Gerards, Arnon P Kater, Eleonora L Swart and Matthijs L Becker in Journal of Oncology Pharmacy Practice
