# Supplementary material for: The effect of a Clinical Decision Support System on the frequency of dose adjustments of anticancer drugs in case of renal or hepatic dysfunction
Source: J Oncol Pharm Pract. 2021 Jun 10;28(5):1063–9. doi: 10.1177/10781552211019453 (PMC10288388; doi:10.1177/10781552211019453)
Supplement: sj-pdf-1-opp-10.1177_10781552211019453 - Supplemental material for The effect of a Clinical Decision Support System on the frequency of dose adjustments of anticancer drugs in case of renal or hepatic dysfunction [file sj-pdf-1-opp-10.1177_10781552211019453.pdf]

Appendix 1: dose adjustment per antineoplastic agent

**Antineoplastic agent**	**Liver or kidney dysfunction**	**Before intervention orders (reduction applied in %)**	**After intervention orders (reduction applied in %)**
Bendamustine	Liver	1 (100%)	4 (75%)
Bleomycin	Kidney	N.A.	1 (100%)
Bortezomib	Liver	N.A.	N.A.
Cisplatine	Kidney	11 (45.5%)	14 (28.6%)
Cyclofosfamide	Both	1 (100%)	2 (50%)
Cytarabine	Both	20 (0%)	19 (15.8%)
Daunorubicin	Liver	1 (0%)	3 (33,3%)
Doxorubicin	Liver	2 (50%)	4 (100%)
Epirubicin	Liver	2 (0%)	1 (100%)
Etoposide	Both	10 (30%)	18 (38.9%)
Fludarabine	Kidney	1 (100%)	3 (33.3%)
Idarubicin	Both	5 (40%)	3 (33.3%)
Ifosfamide	Kidney	N.A.	N.A.
Irinotecan	Liver	4 (25%)	5 (80%)
Melphalan	Kidney	8 (75%)	9 (55.1%)
Methotrexate	Both	N.A.	N.A.
Oxaliplatin	Kidney	2 (50%)	N.A.
Pemetrexed	Kidney	3 (0%)	4 (0%)
Topotecan	Kidney	N.A.	N.A.
Vinblastine	Liver	N.A.	2 (50%)
Vincristine	Liver	2 (0%)	7 (42.9%)
Total:		73 (20.4%)	99 (34.3%)

Dose adjustment per antineoplastic agent. Due to the small amount of orders, no statistic test can be performed for an individual agent. N.A. the agent is not prescribed for a patient with liver or kidney dysfunction during this study period.
